# Preclinical efficacy of carfilzomib in *BRAF*‐mutant colorectal cancer models

**DOI:** 10.1002/1878-0261.13595

**Published:** 2024-02-13

**Authors:** Federica Maione, Daniele Oddo, Federica Galvagno, Chiara Falcomatà, Marta Pandini, Marco Macagno, Valeria Pessei, Ludovic Barault, Chiara Gigliotti, Alessia Mira, Giorgio Corti, Simona Lamba, Chiara Riganti, Barbara Castella, Massimo Massaia, Roland Rad, Dieter Saur, Alberto Bardelli, Federica Di Nicolantonio

**Affiliations:** ^1^ Department of Oncology University of Torino Torino Italy; ^2^ Candiolo Cancer Institute FPO‐IRCCS Candiolo Italy; ^3^ Institute of Molecular Oncology and Functional Genomics School of Medicine, Technical University of Munich Munich Germany; ^4^ Center for Translational Cancer Research (TranslaTUM), School of Medicine Technical University of Munich Munich Germany; ^5^ Tumor Microenvironment Unit Istituto di Ricovero e Cura a Carattere Scientifico Humanitas Research Hospital Milan Italy; ^6^ Department of Biomedical Sciences Humanitas University Milan Italy; ^7^ Laboratory of Blood Tumor Immunology (LBTI), Molecular Biotechnology Center “Guido Tarone” (MBC) University of Turin Turin Italy; ^8^ SC Ematologia Azienda Ospedaliera S. Croce e Carle Cuneo Italy; ^9^ German Cancer Consortium Heidelberg Germany; ^10^ Department of Internal Medicine II, Klinikum rechts der Isar Technische Universität München Munich Germany; ^11^ IFOM ETS The AIRC Institute of Molecular Oncology Milan Italy

**Keywords:** *BRAF* mutant colorectal cancer, endoplasmic reticulum stress, immune microenvironment, immunogenic cell death, oncogene, proteasome inhibitors

## Abstract

Serine/threonine‐protein kinase B‐raf (*BRAF*) mutations are found in 8–15% of colorectal cancer patients and identify a subset of tumors with poor outcome in the metastatic setting. We have previously reported that *BRAF‐*mutant human cells display a high rate of protein production, causing proteotoxic stress, and are selectively sensitive to the proteasome inhibitors bortezomib and carfilzomib. In this work, we tested whether carfilzomib could restrain the growth of *BRAF‐*mutant colorectal tumors not only by targeting cancer cells directly, but also by promoting an immune‐mediated antitumor response. In human and mouse colorectal cancer cells, carfilzomib triggered robust endoplasmic reticulum stress and autophagy, followed by the emission of immunogenic‐damage‐associated molecules. Intravenous administration of carfilzomib delayed the growth of *BRAF‐*mutant murine tumors and mobilized the danger‐signal proteins calreticulin and high mobility group box 1 (HMGB1). Analyses of drug‐treated samples revealed increased intratumor recruitment of activated cytotoxic T cells and natural killers, concomitant with the downregulation of forkhead box protein P3 (Foxp3)^+^ T‐cell surface glycoprotein CD4 (CD4)^+^ T cells, indicating that carfilzomib promotes reshaping of the immune microenvironment of *BRAF‐*mutant murine colorectal tumors. These results will inform the design of clinical trials in *BRAF‐*mutant colorectal cancer patients.

## Graphical abstract

We show that the endoplasmic reticulum stress elicited by carfilzomib in human and murine *BRAF* mutant colorectal cancer cells promotes the activation of immunostimulant danger signals, and the mobilization of immunogenic cell death markers. In *BRAF*‐mutant murine colorectal tumors, carfilzomib promotes reshaping of the immune microenvironment by recruiting activated cytotoxic T cells and natural killers while impairing regulatory T cells.

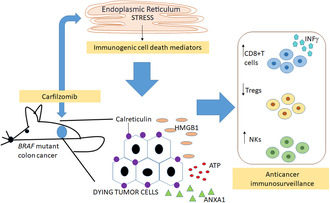

**Abbreviations**
ANXA1: annexin A1
BRAF: serine/threonine‐protein kinase B‐raf
CD4: T‐cell surface glycoprotein
CRC: colorectal cancer
DAMPs: damage‐associated molecular patterns
DAPI: diamidino‐2‐phenylindole
DMEM: Dulbecco's modified eagle medium
DMSO: dimethyl sulfoxide
ELISA: Enzyme‐Linked Immunosorbent Assay
ER: endoplasmic reticulum
FoxP3: forkhead box protein P3
HMGB1: high mobility group box 1
i.v: intravenous
ICD: immunogenic cell death
INFγ: interferon γ
mCRC: metastatic colorectal cancer
MHC1: majority of class I
MSS: microsatellite stable
NK: natural killer
PAF: paraformaldehyde
p‐elF2α: phosphorylated eIF2α
Tregs: T regulatory cells
UPR: unfolded protein response

## 1 Introduction

Colorectal cancer (CRC) is the fourth most common malignancy and the second most frequent cause of cancer‐related death worldwide [1]. Among CRCs, the missense mutation p.V600E in *BRAF* gene occurs in about 8–15% of the cases, identifying a distinct subset of tumors with aggressive phenotype and poor outcome in the metastatic setting [2, 3]. The use of chemotherapy or targeted agents showed a limited benefit in *BRAF* mutant metastatic CRC (mCRC) patients, with the exception of combined anti‐BRAF/EGFR inhibitors in the second/third line setting, which however only achieved an overall survival of about 9 months [4]. A phase II study is currently testing BRAF and EGFR targeted agents in combination with immune checkpoint blockade with the goal of achieving more durable responses in the limited subset of tumors co‐harboring *BRAF* mutations and microsatellite instability [5]. Novel therapeutic strategies are needed to improve the therapeutic outcome of *BRAF* mutant mCRC patients, particularly for those displaying microsatellite stable (MSS) status.

Recent studies have reported that the immune microenvironment in *BRAF* mutated tumors might lead to resistance to conventional therapies through regulation of the composition of immune cell infiltration and chemokines [6, 7]. Therefore, therapeutic strategies targeting both CRC cells and the tumor microenvironment could be key to enhance the recognition of the tumor cells by the immune system and achieve long‐lasting responses in *BRAF* mutant bowel cancer patients.

In addition to their direct cytotoxic effect on neoplastic cells, a subset of chemotherapeutic drugs, such as oxaliplatin and anthracyclines exert their antitumor activity also by an indirect immune‐dependent mechanism [8]. Pre‐mortem activation of specific stress pathways in dying tumor cells causes the release or exposure of molecules that serve as danger signals, defined as damage‐associated molecular patterns (DAMPs). In turn, DAMPs exert robust immunostimulatory effects through the binding to pattern recognition receptors expressed by immune cells. This peculiar cell death process is defined as immunogenic cell death (ICD) [9, 10, 11]. ICD involves the activation of at least two pathways: (a) the endoplasmic reticulum (ER) stress response, a pathway activated upon perturbation of the ER homeostasis, which causes exposure of the chaperone calreticulin on the cell surface [12, 13]; (b) autophagy, a lysosomal degradation pathway, which causes ATP release in the extracellular environment. Calreticulin acts as an ‘eat‐me’ signal helping the dendritic cells to phagocyte dying cancer cells, while ATP operates as a chemoattractant by interacting with the purinergic receptors of dendritic cells [14]. In addition, ICD is characterized by the release of high mobility group box 1 (HMGB1), a nuclear protein that elicits dendritic cell maturation through the activation of the Toll‐like receptor‐4 [15]. Finally, annexin A1 (ANXA1) is released in the extracellular microenvironment and binds the formyl peptide receptor 1 expressed on dendritic cells, contributing to their correct positioning in proximity of dying tumor cells [16]. The emission of the aforementioned DAMPs is mainly responsible for determining whether cell death will be immunogenic [17]; the failure of cancer cells to emit one or more of these DAMPs ultimately compromises the immunogenicity of the dying cells [8]. Indeed, a complex signaling network elicited by DAMPs governs communication between dying cancer cells, and the appropriately disposed immune system. These preclinical observations are supported by several clinical studies that demonstrated the beneficial immunostimulatory effects of ICD induction by chemotherapy [18], underlying the importance of investigating ICD inducers for clinical applications.

The proteasome inhibitor bortezomib has recently been shown to promote the activation of ICD‐related pathways in multiple myeloma [19, 20]. Indeed, inhibition of proteasome activity leads to the accumulation of unfolded or misfolded proteins in the ER, thus inducing the so‐called ER stress, a particular stressful condition caused by the imbalance between the demand for protein folding and the ability of the ER to sustain it [21, 22].

Interestingly, a high proteasomal activity and accumulation of unfolded and ubiquitinated proteins has been found as a consequence of increased protein synthesis elicited by oncogenic BRAF V600E signaling [23]. We and others have proposed that these cellular peculiarities create a status of dependency on the proteasomal degradation machinery in human BRAF mutant CRC cells, rendering them selectively sensitive to the proteasome inhibitors carfilzomib and bortezomib [24, 25].

Here we report that the irreversible second‐generation proteasome inhibitor carfilzomib [23] activates autophagy and ER stress pathways in *BRAF* mutant preclinical models of CRC. Carfilzomib mediates mobilization of DAMPs responsible for activating the immune system against cancer cells. Finally, we find that carfilzomib treatment in immunocompetent models of *BRAF* mutant CRC enhances the activation of cytotoxic T cells and natural killers (NKs). The discovery that carfilzomib can reshape the microenvironment of *BRAF* mutant murine CRCs paves the way for testing this drug in combination with other immunomodulating therapies in *BRAF* mutant mCRC patients.

## 2 Materials and methods

### 2.1 Cell lines and cell authentication

WiDr (RRID:CVCL_2760) cells were kindly provided from Dr Renè Bernards (Amsterdam, The Netherlands) in July 2011. HROC24 (RRID:CVCL_S853) were shared by Michael Linnebacher (Rostock, Germany) in September 2011. JVE127 (RRID:CVCL_EG22) and KP363T (RRID:CVCL_EG34) were obtained from Leiden University Medical Center (Leiden, The Netherlands) in 2016. Murine VBC9 and VPFB6 were a gift of Prof. Roland Rad (Munich, Germany) in October 2017.

VBC9 and VPFB6 are cell lines derived from transgenic mouse models of CRC carrying the paralogue *Braf* mutation V637E [18]. The mutation of *Braf*  coexists with the *Tp53 R171H* and *Cdkn2a* ^−/+^ alterations in VBC9 and VPFB6 cells, respectively. Since both murine cell lines were characterized by a low engraftment ability in C57/BL6 immunocompetent mice, we proceeded with an immunoediting protocol. Specifically, both VPFB6 and VBC9 cells were first grown in immunocompromised mice to generate tumors before being injected into immunocompetent models. Afterwards, we set up a cyclic protocol which included four steps: cell injection, tumor growth, cell derivation from tumor pieces and re‐injection in immunocompetent mice. Through this experimental approach, we obtained VBC9‐ and VPFB6‐ derived cell lines characterized by a high engraftment ability in C57/BL6 immunocompetent mice and which were used for all the experiments of this work. Whole exome sequencing confirmed the presence of *Braf* mutation in both murine cell lines and their MSS status.

The *BRAF* mutant human WiDr, KP363T, JVE127 CRC cell lines were grown in RPMI‐1640, HROC24 in Dulbecco's Modified Eagle Medium (DMEM) F12; murine VBC9 and VPFB6 cell lines were cultured in DMEM high glucose. All cell culture media were supplemented with 10% (v/v) fetal bovine serum, 2 mm L‐glutamine and antibiotics (100 U·mL^−1^ penicillin, 100 mg·mL^−1^ streptomycin and 25 μg·mL^−1^ amphotericin B) to avoid contamination. Cells were maintained under standard culture condition (37 °C, 5% CO2, > 90% humidity) and were tested to exclude Mycoplasma contamination using the VenorGeM Classic Kit (Minerva Biolab, Berlin, Germany), thus ensuring that all the *in vitro* experiments were performed on mycoplasma‐free cells.

### 2.2 Cell genetic identity authentication

The genetic identity of human cell lines was authenticated using the PowerPlex^®^ 16 HS System (Promega, Madison, WI, USA), through Short Tandem Repeats (STR) at 16 different loci (D5S818, D13S317, D7S820, D16S539, D21S11, vWA, TH01, TPOX, CSF1PO, D18S51, D3S1358, D8S1179, FGA, Penta D, Penta E, and amelogenin). The results are listed in Table S1. STR profiling of *BRAF* mutant murine cell lines was performed as a service by LGC STANDARDS srl. Amplicons from multiplex PCRs were separated by capillary electrophoresis (3730 DNA Analyzer, Applied Biosystems, Waltham, MA, USA). The results are listed in Table S2.

### 2.3 Drugs

Cisplatin and oxaliplatin were purchased from Accord Healthcare UK (London, UK). Carfilzomib was purchased from MedChemExpress (Monmouth Junction, NJ, USA); chloroquine and tunicamycin from Sigma‐Aldrich (St. Louis, MO, USA).

### 2.4 Drug proliferation assays

Cell proliferation experiments were carried out in 24‐well plates in duplicate. A total of either 8000 cells for WiDr, KP363T, JVE127, HROC24 or 4000 cells for VBC9 and VPFB6 were seeded in 450 μL complete growth medium. At 24 h post‐seeding, 50 μL of serum‐free medium with or without carfilzomib was manually added to the cells. Cells were fixed with 4% (w/v) paraformaldehyde (PAF) and stained with 1% crystal violet‐methanol solution (Sigma‐Aldrich) after 12–14 days. All assays were performed independently two times.

### 2.5 Drug cytotoxicity assay

For the cytotoxicity assay cells were seeded into 96‐well black optical‐bottom plates (Nunc, Life Technologies, Waltham, MA, USA) in 50 μL growth culture medium (4000 cells per well for WiDr, KP363T, JVE127, HROC24, 1000 cells per well for VBC9 and VPFB6). At 24 h post‐seeding, 50 μL of serum‐free medium with or without carfilzomib (both 100 nm) was manually added to the cells. After 72 h of treatment, the CellTox Green cytotoxicity assay (Promega) was performed according to manufacturer instructions and fluorescence was read by TECAN Spark^®^ 10M plate reader at 535 nm. The experiments were carried out in duplicate. Subsequently, the number of viable cells for each well was quantified by CellTiter‐Glo Luminescent Assay (Promega). Data were first normalized to the number of cells and after to untreated control. Analysis and graph were performed using graphpad prism Software (Boston, MA, USA).

### 2.6 Proteasome inhibition assay

Proteasome inhibition was assayed using the ProteasomeGlo™ Chymotrypsin‐Like Cell‐Based Assay (Promega) following the manufacturer's instructions. Cells were plated in 96 well plates (8000 cells per well for WiDr; 5000 cells per well for VBC9 and VPFB6), incubated for 16 h, and then exposed to 100 nm of carfilzomib. Proteasome activity was measured at different time points (1, 2, 4, 6, 16 or 24 h) upon carfilzomib treatment, comparing the proteasome inhibition to the untreated condition. The experiments were repeated two times.

### 2.7 Detection of apoptosis by flow cytometer

Apoptosis was assessed by means of the Annexin V‐fluorescein isothiocyanate (FITC) detection kit (eBioscience, Waltham, MA, USA) according to the manufacture's procedures. The analysis was repeated three times. Specifically, cells treated in 6‐well plate were collected, washed in PBS and resuspended in 100 μL binding buffer containing FITC‐conjugated AnnexinV antibody. Samples were then incubated in the dark for 15 min before adding 400 μL binding buffer supplemented with propidium iodide (PI). Data were acquired on a CyAn™ ADP cytofluorometer (Beckman Coulter, Brea, CA, USA).

### 2.8 Western blot analysis: Drug treatments and antibodies

Before biochemical analysis, cells were grown in absence or presence of carfilzomib 100 nm, chloroquine 3 μm or tunicamycin 1 mg·mL^−1^ for the times indicated in figure legends (*n* = 3). Total cellular proteins were extracted by lysing cells in boiling Laemmli buffer (1% SDS, 50 mm Tris–HCl [pH 7.5], 150 mm NaCl). Extracts were clarified by centrifugation, normalized with the BCA Protein Assay Reagent kit (Thermo Scientific, Waltham, MA, USA). Proteins were separated on 4–12% NuPAGE Novex Bis‐Tris gels in the NuPAGE MES SDS Running Buffer (Invitrogen, Waltham, MA, USA) and electrotransferred to 0.45 μm polyvinylidene fluoride (PVDF) membranes (Bio‐Rad, Hercules, CA, USA). Western blot detection was performed with enhanced chemiluminescence system (Amersham, Buckinghamshire, UK) and peroxidase‐conjugated secondary antibodies (Sigma‐Aldrich). Chemiluminescent signal was acquired by ChemiDoc Imaging System (Biorad). The following primary antibodies were used (all from Cell Signaling Technology [Danvers, MA, USA] and at a dilution 1 : 1000, except where otherwise indicated): anti‐ubiquitin (Santa Cruz Biotechnology, Dallas, TX, USA), anti‐PARP, anti‐LC3, anti‐BIP, anti‐eIF2α, anti‐phospho‐eIF2α, anti‐CHOP, anti‐tubulin (1 : 5000), anti‐actin (Santa Cruz Biotechnology 1 : 5000), anti‐HMGB1.

### 2.9 Detection of ICD markers

Human and murine CRC cells were seeded into 6‐well plates and treated with carfilzomib (100 nm), cisplatin (50 μm), oxaliplatin (50 μm) for 24 h before ICD markers detection. All the experiments were carried out at least three times.

The translocation of calreticulin to the cell surface was assessed by flow cytometry. Specifically, cells were washed twice with PBS, detached with accutase (Sigma‐Aldrich) and cell suspensions were stained for 30 min with a non‐immune mouse IgG1 isotype phycoerythrin‐conjugated antibody (Abcam, Cambridge, UK), or with anti‐calreticulin phycoerythrin‐conjugated antibody (Abcam) diluted in cold blocking buffer (2% fetal bovine serum in PBS). Cells were washed and resuspended in PBS containing 4,6‐diamidino‐2‐phenylindole (DAPI) before acquisition on a CyAn™ ADP cytofluorometer (Beckman Culture). In order to exclude dead cells from the analysis, the fluorescence intensity of stained cells was gated on DAPI‐negative cells. In order to assess the percentage of calreticulin‐positive DAPI‐negative cells, data were analyzed using flowjo.

ATP release was evaluated normalizing the luminescence value (proportional to the ATP content present in the media) to the crystal violet absorbance (proportional to cell confluence). ATP content was measured by CellTiter‐Glo Luminescent Assay (Promega): 90 μL of the cell culture medium were transferred in a white 96‐well plate in which 10 μL of CellTiter‐Glo were added before measurement of luminescence. In order to assess cell confluence, cells attached to the plate were fixed with PAF 4% and stained with crystal violet. Crystal violet was then solubilized with 33% (w/v) acetic acid and absorbance was quantified at 595 nm. Both luminescence and absorbance were measured by the SPARK M10 (Tecan, Männedorf, Switzerland) plate reader.

To measure the extracellular release of HMGB1, 20 μL of the cells culture medium were boiled, resolved by SDS/PAGE and probe with an anti‐HMGB1 antibody (Sigma‐Aldrich). Blots were pre‐stained with Red Ponceau to check the equal loading of proteins.

ANXA A1 concentrations were measured in the supernatant of cells following the indicated treatment by means of an Enzyme‐Linked Immunosorbent Assay (ELISA) kit (REF MOEB0294 and HUEB0593, Cusabio, Houston, TX, USA), according to the manufacturer's protocol. Absorbance values were measured using SPARK M10 (Tecan) plate reader.

### 2.10 Animal studies

All animal studies were approved by the Ethical Commission of the Candiolo Cancer Institute FPO‐IRCCS and by the Italian Ministry of Health and performed following institutional guidelines and international law and policies (Approval number 811/2019‐PR). The number of mice included in the experiments and the inclusion/exclusion criteria were based on institutional guidelines. We observed tumor size limits in accordance with institutional guidelines. Our protocol limited us to using 6–8‐week‐old female and male C57BL/6J and NOD‐SCID mice. Mice were obtained from Charles River. Animals were housed in individually ventilated cages in the animal facility of the institute under sterile conditions and optimal temperature and humidity. Enrichment devices were employed to increase the animal welfare, and the experiments were conducted according to 3R principles and animal welfare was regularly monitored. Tumor size was measured using the formula: *V* = (*d* ^2^ × *D*)/2 (*d* = minor tumor axis; *D* = major tumor axis) and reported as tumor mass volume (mm^3^, mean ± SEM of individual tumor volume). No statistical methods were used to predetermine sample size.

### 2.11 Mouse treatments

VBC9 or VPFB6 cells (2 × 10^6^ cells per mouse) were subcutaneously inoculated into the right flank of NOD‐SCID (*n* = 10) immunocompromised or C57BL/6 immunocompetent mice (*n* = 10). Once the tumors reached around 20–70 mm^3^ in size (typically after 8–12 days) animals were randomized to receive either vehicle or intravenous (i.v) carfilzomib. Carfilzomib was formulated in an aqueous solution of 10% (w/v) sulfobutylether‐b‐cyclodextrin (Captisol, from CYDEX Pharmaceuticals Inc, Lenexa, KS, USA) and 10 mmol·L^−1^ sodium citrate (pH 3.5). Carfilzomib solutions were diluted daily with vehicle before i.v injections and administered on days 1, 2, 8, 9 at a dose of 4 mg·kg^−1^.

### 2.12 Orthotopic implantation of VPFB6 cells

Six‐week‐old C57BL/6 mice were treated subcutaneously with pre‐operative analgesia (0.5% Meloxicam 5 mg·kg^−1^) and with antibiotic (2.5% Enrofloxacin 10 mg·kg^−1^). Tumor cell implantation was performed as previously described [26]. During all the surgery mice were anesthetized by using 2.5 isoflurane inhalation with 3–5 L·min^−1^ airflow. Once the cecum was exposed CRC cell lines (1 × 10^6^ cells per mouse) were micro‐injected into the cecum submucosa using a glass micro‐capillary. Six weeks later, mice were injected with luciferin (15 mg·mL^−1^) (PerkinElmer, Waltham, MA, USA) and their *in vivo* tumor bioluminescence was analyzed using IVIS^®^ Lumina II imaging system (Perkin Elmer). Then animals were treated with carfilzomib twice a week as previously described (*n* = 5). At the end point of the experiments, mice were injected with luciferin (15 mg·mL^−1^) (PerkinElmer), sacrificed, and their organs extracted for metastasis formation using IVIS^®^ Lumina II imaging system. Following IVIS imaging, the guts were embedded in OCT and freshly frozen for histological analysis.

### 2.13 *Ex vivo* – tumor processing and phenotyping of the tumor immune infiltrate

C57BL/6 immunocompetent were treated intravenously with carfilzomib or vehicle (*n* = 15) for 2 weeks. Mouse tumors were cut into small pieces, disaggregated using Miltenyi Biotec mouse tumor dissociation kit and gentleMACS™ Octo Dissociator following the manufacturer's instructions. Tumor homogenates were filtered through 70 μm. Cells were stained with specific antibodies and DAPI to exclude dead cells. The same methods have been previously described in Magrì et al in 2020 [27] Phenotype analysis was performed with the following antibodies purchased from Bio‐Legend (San Diego, CA, USA) or Becton Dickinson (Wokingham, UK): anti‐CD45‐PerCp (30F11), anti‐CD11b‐APC (M1/70), anti‐CD3‐PE/Cy7 (17A2), anti‐CD4‐FITC (RM4‐5), anti‐CD8‐PE or FITC (YTS156.7.7), anti‐F4/80‐APC (BM8), anti‐CD49b‐PE (DX5), anti‐CD44‐APC (IM7), anti‐CD69‐PE (H1.2F3), anti‐CD62L‐Pe/Cy7 (MEL‐14), anti‐CD11c‐FITC (N418), anti‐CD28‐PE (37.51), anti‐CD25‐APC (PC61), anti‐CD127‐Pe/Cy7 (A7R34), anti‐FoxP3‐PE (MF‐14). For FoxP3 staining of tumor‐infiltrating lymphocytes, cells were isolated and stained with surface antibodies for 30 min, and then fixed and permeabilized using the FoxP3 Fix/Perm Buffer set (Bio‐Legend). Cells were then stained with FoxP3‐PE (Bio‐Legend). For IFNγ staining, cells were stimulated 6 h with cocktail as indicated in the protocol and then stained for extracellular markers, cell permeabilization was performed with the BD kit and then intracellularly stained with IFNγ (XMG1.2 Bio‐Legend). All flow cytometry was performed using the FACS Dako instrument and analyzed using flowjo software (TreeStar, Inc., Culver City, CA, USA).

### 2.14 Tissue immunofluorescence

Confocal analysis was performed on fresh frozen tissues. In brief, tumor samples were included in Killik OCT (Bio‐Optica, Milan, Italy), serially cut (10 μm), and fixed using zinc fixative solution. Then, samples were incubated for 1 h in blocking buffer (Dako Solution, Troy, MI, USA) and incubated overnight with the following primary antibodies: anti‐CD4‐FITC (RM4‐5), anti‐CD8‐PE (YTS156.7.7), anti‐FoxP3‐PE (MF‐14), anti‐IFNγ‐FITC (XMG1.2 Bio‐Legend), anti‐calreticulin (Thermo Fisher Scientific) and anti‐HMGB1 (Thermo Fisher Scientific). For the detection of the unconjugated antibodies, anti‐rabbit Alexa Fluor 555 (Thermo Fisher Scientific) was used. Nuclei were stained with DAPI. Slides were then mounted using fluorescence mounting medium (Dako) and analyzed using a confocal laser scanning microscope (TCS SPE II; Leica, Wetzlar, Germany). Images quantification was performed by means of leica lasaf software considering 5 field per samples for at least 5 samples per conditions.

### 2.15 Statistics

Statistical analyses were performed using graphpad prism software. All data are presented as either mean ± SD or ± SEM (as indicated in figure legends). Mann–Whitney test was performed for cell death assessment, calreticulin translocation on cell surface, ATP release and to assess whether the difference in tumor growth was significant among carfilzomib treated versus vehicle animals. Symbols for statistical comparison are *P < 0.05; **P < 0.01; ***P < 0.005. NS; not significant. The number of replicates and sample size for *in vivo* experiments were limited according to the requirements of the Italian Ministry of Health. Animal studies were performed in accordance with institutional guidelines and international law and policies.

## 3 Results

### 3.1 Carfilzomib is cytotoxic in human and murine *BRAF* mutant CRC cells

To investigate the ability of the proteasome inhibitor carfilzomib in mediating both autonomous and non‐autonomous cell death, we firstly assessed its cytotoxic effect on a panel of both human and murine CRC cell lines. We employed four human cell lines (JVE127, HROC24, WiDr and KP363T) and two murine MSS CRC cell lines (VBC9 and VPFB6) carrying the paralogue *BRAF* mutation V637E (from here referred to as V600E) [18]. VPFB6 cells concomitantly carried the *TP53* R172H homozygous mutation, whereas a *CDKN2A* stop‐gain variant was present in VBC9. These genomic alterations were experimentally introduced in the colon of transgenic mice to increase the tumorigenicity of the BRAF mutant expressing cells [19, 20, 28].

**Fig. 1:**
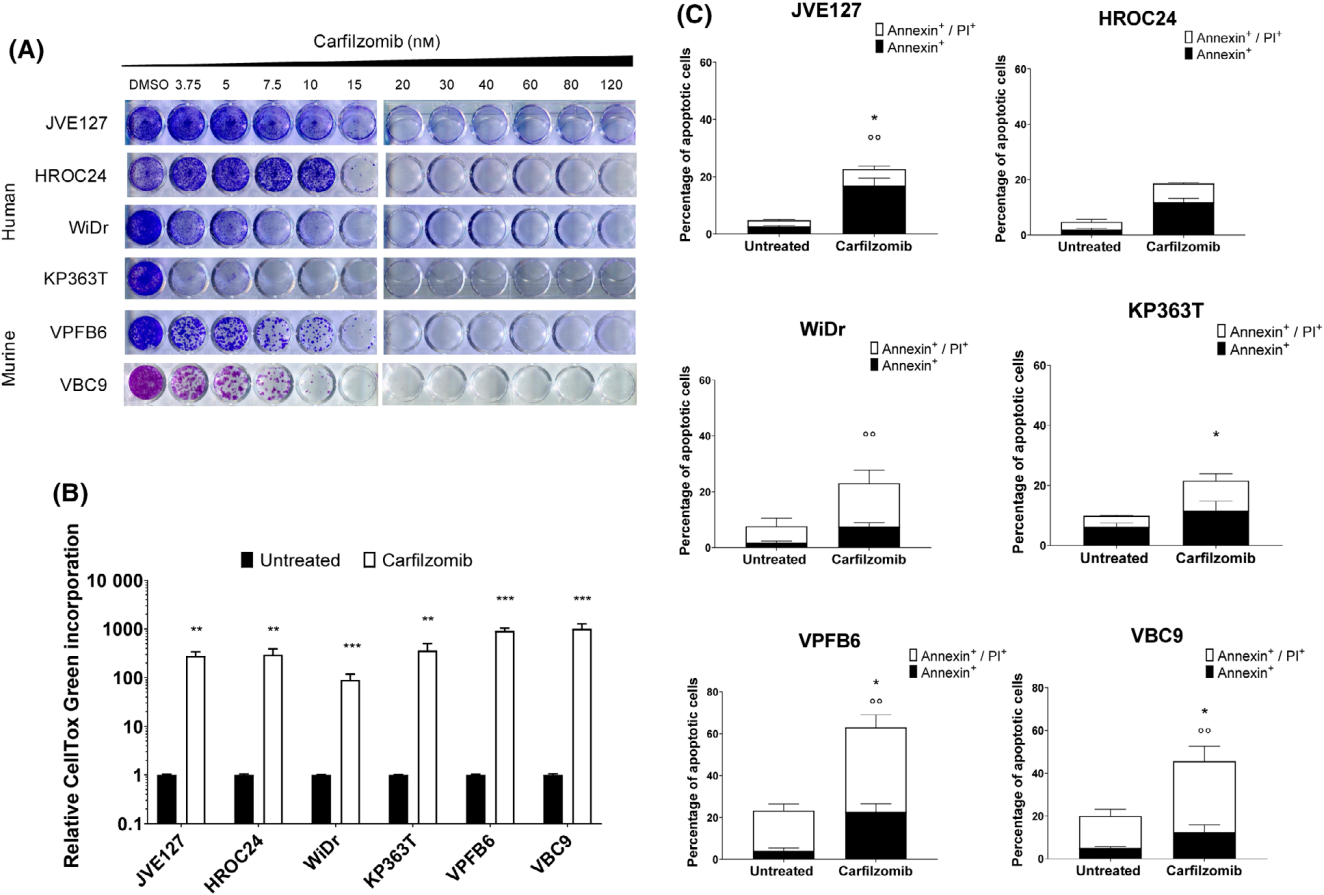
Carfilzomib impairs cell proliferation, promoting apoptosis in both human and murine *BRAF* mutant CRC cell lines. (A) Long‐term colony‐forming assay of *BRAF* mutant CRC cell lines treated for 5–10 days in the absence or presence of carfilzomib at the indicated nanomolar concentrations. Results are representative of 2independent observations. Dimethyl sulfoxide (DMSO) treated cells were used as proliferation control. (B) CellTox Green cytotoxicity assay performed on human and murine cell lines. Cells were cultured for 72 h in the absence or presence of carfilzomib (100 nm). Histograms and error bars indicate mean ± SEM of 3 independent experiments. Statistical differences were determined using the Mann–Whitney *U* test. **P < 0.01, ***P < 0.001 as compared to untreated control cells. (C) Flow cytometric analysis of Annexin V/PI labelling of human and murine cells untreated or treated for 24 h with carfilzomib (100 nm). The graph shows early (black) annexin V‐positive and late (white, both) annexin and PI (Propidium Iodide) ‐positive apoptotic cells. Data and error bars indicate mean ± SEM, and are representative of 3 independent experiments. Statistical differences were determined using the Mann–Whitney *U* test. *P < 0.01, °°*P* < 0.01 as compared to untreated control cells. Symbol * refers to Ann^+^/PI^+^; symbol ° refers to Ann^+^.

**Fig. 2:**
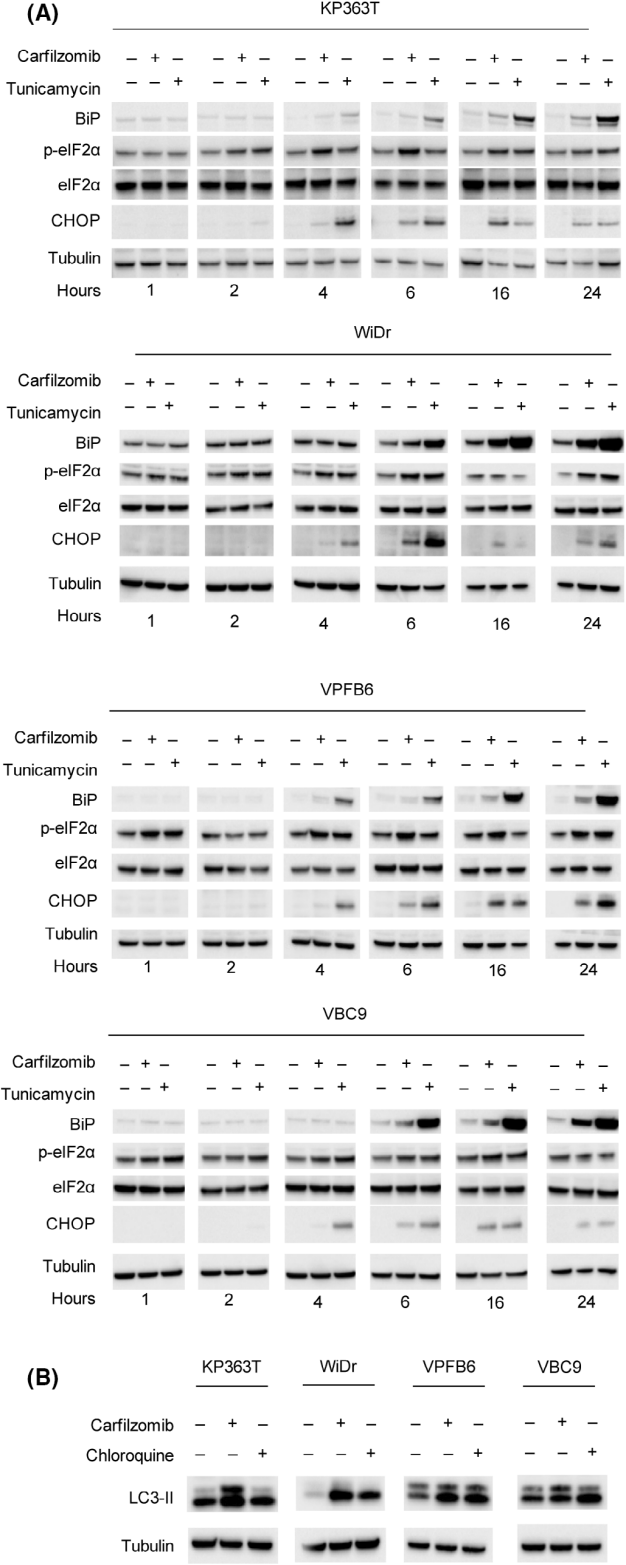
Carfilzomib triggers ER stress response and autophagy in *BRAF* mutant CRC cell lines. (A) Expression of ER stress markers in human and murine CRC cell lines untreated or treated with carfilzomib (100 nm) or tunicamycin (1 μg·mL^−1^). Proteins/cellular lysates were collected at different time points (1, 2, 4, 6, 16, 24 h). (B) Lipidated LC3‐II protein levels after 24 h of treatment with carfilzomib (100 nm) or autophagy inhibitor chloroquine (3 μm). An antibody against tubulin was used as loading control. The analysis was replicated twice for each cell line.

*In vitro* treatment with carfilzomib reduced the proteasome chymotrypsin like activity and increased ubiquitinated protein accumulation in human and mouse *BRAF* mutant CRC (Fig. S1A,B). Carfilzomib decreased *BRAF* mutant tumor cell proliferation in a dose‐dependent manner in a colony‐forming assay (Fig. 1A). Drug treatment also induced cell death in all treated cells compared with controls (Fig. 1B). In addition, cytofluorimetric analysis of annexin V exposure on the cell surface revealed that carfilzomib induced apoptosis already 24 h after treatment (Figs 1C and 2). Together these results show that carfilzomib is cytotoxic in both human and murine *BRAF* mutant CRC cell lines *in vitro*.

### 3.2 Carfilzomib induces ER stress and autophagy in *BRAF* mutant CRC cells

Proteasome inhibitors have been reported to cause the accumulation of misfolded proteins, leading to ER stress and thereby eliciting the unfolded protein response (UPR) pathway in multiple myeloma [29]. To test whether carfilzomib could induce ER stress in CRC models, we selected *BRAF* mutant murine and human cell lines that were most sensitive to carfilzomib. We examined the expression levels of several proteins commonly involved in UPR [30]. Among the proteins implicated in this signal transduction pathway, we focused on the following ER key regulators: (a) BiP, an ER chaperone acting as a sensor of protein accumulation; (b) eIF2α, which is a repressor of protein synthesis involved in the translocation of calreticulin on the cell surface; (c) CHOP, a marker of unresolved ER stress and an apoptosis mediator. Tunicamycin was used as positive control [31]. CHOP expression and the phosphorylation of eIF2α (p‐elF2α) levels significantly increased at 4 and 6 h after carfilzomib, respectively [13]. An evident increase of BiP expression was seen after 24 h of drug treatment (Fig. 2A). Altogether, these results indicate that proteasome inhibition can elicit UPR pathways in BRAF mutant CRC cells. A consequence of the UPR is the elimination of the misfolded proteins through autophagy, a degradation mechanism involved in the maintenance of cellular homeostasis [32, 33]. Previous findings revealed that ER stress can either inhibit or stimulate autophagy [34]. To investigate whether the ER stress induced by carfilzomib could influence autophagy, we measured the expression of the autophagy‐related protein LC3‐II. The autophagy inhibitor chloroquine was used as positive control to decrease the autophagosome‐lysosome fusion, resulting in an increase in the cellular level of LC3‐II [35, 36]. Although LC3‐II could be detected already in basal conditions in *BRAF* mutant cells, drug treatment increased LC3‐II protein levels, indicating that carfilzomib may be able to modulate autophagy (Fig. 2B). Previous studies show that autophagy promoted by the proteasome inhibitor bortezomib can mediate either cell survival or cell death, depending on the cell type [37, 38]. To elucidate the effect of carfilzomib on *BRAF* mutant CRC cells on cell survival we performed a proliferation assay, and we found that the co‐treatment with a sub‐lethal dose of chloroquine significantly improved the killing effect of carfilzomib in *BRAF* mutant cells (Fig. S3). These findings collectively indicate that autophagy induced by carfilzomib promotes *BRAF* mutant CRC cell survival, thus decreasing its cytotoxic effect.

### 3.3 Carfilzomib promotes the emission of immunostimulant danger signals

**Fig. 3:**
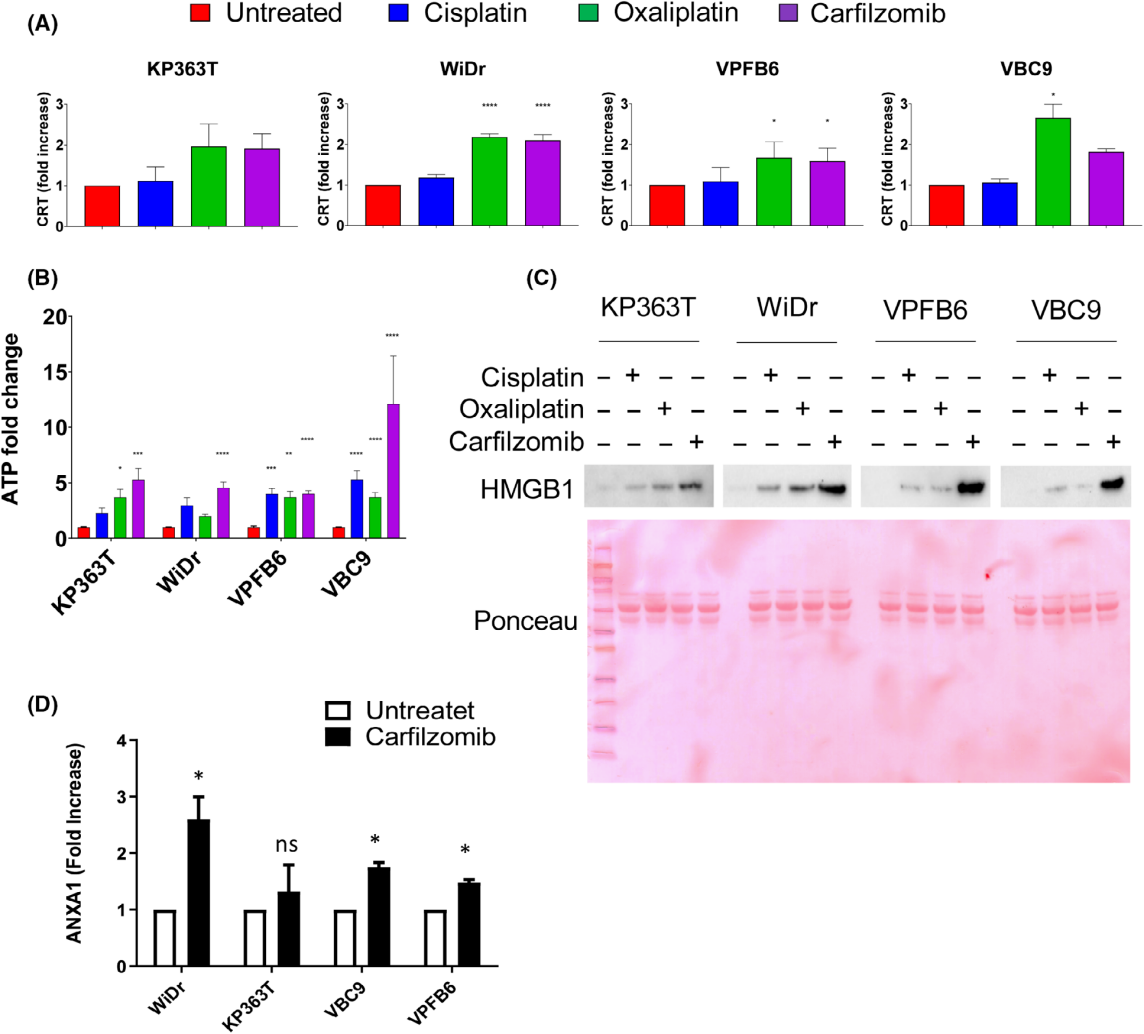
Mobilization of DAMPs by carfilzomib in *BRAF* mutant CRC lines. (A) Flow cytometry analysis of calreticulin exposure on the surface of CRC cells in the absence of treatment or upon 24 h stimulation with cisplatin (50 μm), oxaliplatin (50 μm) or carfilzomib (100 nm). The fold increase of calreticulin exposure was calculated by normalizing the geometric mean fluorescence intensity of calreticulin of each sample on the relative control. Error bars are representative of ±SD of 3 independent experiments. (B) Carfilzomib‐induced ATP release was detected in cell supernatant after 16 h of treatment with cisplatin (50 μm), oxaliplatin (50 μm) or carfilzomib (100 nm) by luminescence assay. Error bars are representative of ±SD of 3 independent experiments. (C) Western blot analysis showing HMGB1 levels in cell supernatant after 16 h treatment with cisplatin, oxaliplatin or carfilzomib in CRC cells. Red ponceau staining was used as loading control. The analysis was replicated at 3 times for each cell line. (D) ANXA1 levels in the supernatant after an overnight treatment with carfilzomib detected by ELISA. Data represent mean ± SD of 3 independent experiments. Statistical differences were determined using the Mann–Whitney *U* test. *P < 0.05, **P < 0.01, ***P < 0.001, ****P < 0.0001 as compared to untreated control cells.

ER stress and autophagy pathways are tightly related to the process of ICD. We hypothesized that carfilzomib‐induced cell death could cause the exposure of danger signals, thus evoking anticancer immune responses. To test this, we assessed calreticulin translocation on the cell surface after carfilzomib treatment. Of note, among platinum‐based chemotherapeutic drugs, only oxaliplatin is able to trigger immunogenic cell death in preclinical settings, while cisplatin is not an ICD inducer. Therefore, in our study oxaliplatin and cisplatin were used as negative and positive controls, respectively. By flow cytometry analysis, a significant increase of calreticulin was found in both WiDr and VPFB6 treated with carfilzomib compared with their respective control. Mobilization of calreticulin on the surface, even though not statistically significant, was observed also in KP363T and VBC9 in the presence of carfilzomib (Fig. 3A). A significant accumulation of extracellular ATP was observed in all cell lines treated with carfilzomib (Fig. 3B). We also found increased HMGB1 protein levels in the supernatant of cells treated with carfilzomib compared with all the other tested conditions (Fig. 3C). Notably, in this experiment we could not use intracellular proteins such as tubulin, actin, vinculin, as loading controls since we analyzed the cell supernatant. Instead, we employed Ponceau staining that is conventionally accepted to monitor protein loading in case of supernatant proteins. Finally, by ELISA immunoassay we found a statistically significant increase of ANXA1 release in the supernatant of carfilzomib‐treated VPFB6, VBC9 and WiDr cells (Fig. 3D). These findings reveal that carfilzomib can promote the mobilization of DAMPs in both human and murine *BRAF* mutant CRC cells, most likely in response to ER stress and autophagy.

### 3.4 Carfilzomib restrains tumor growth and mobilizes ICD markers in immunocompetent mice bearing *BRAF* mutant colorectal tumors

**Fig. 4:**
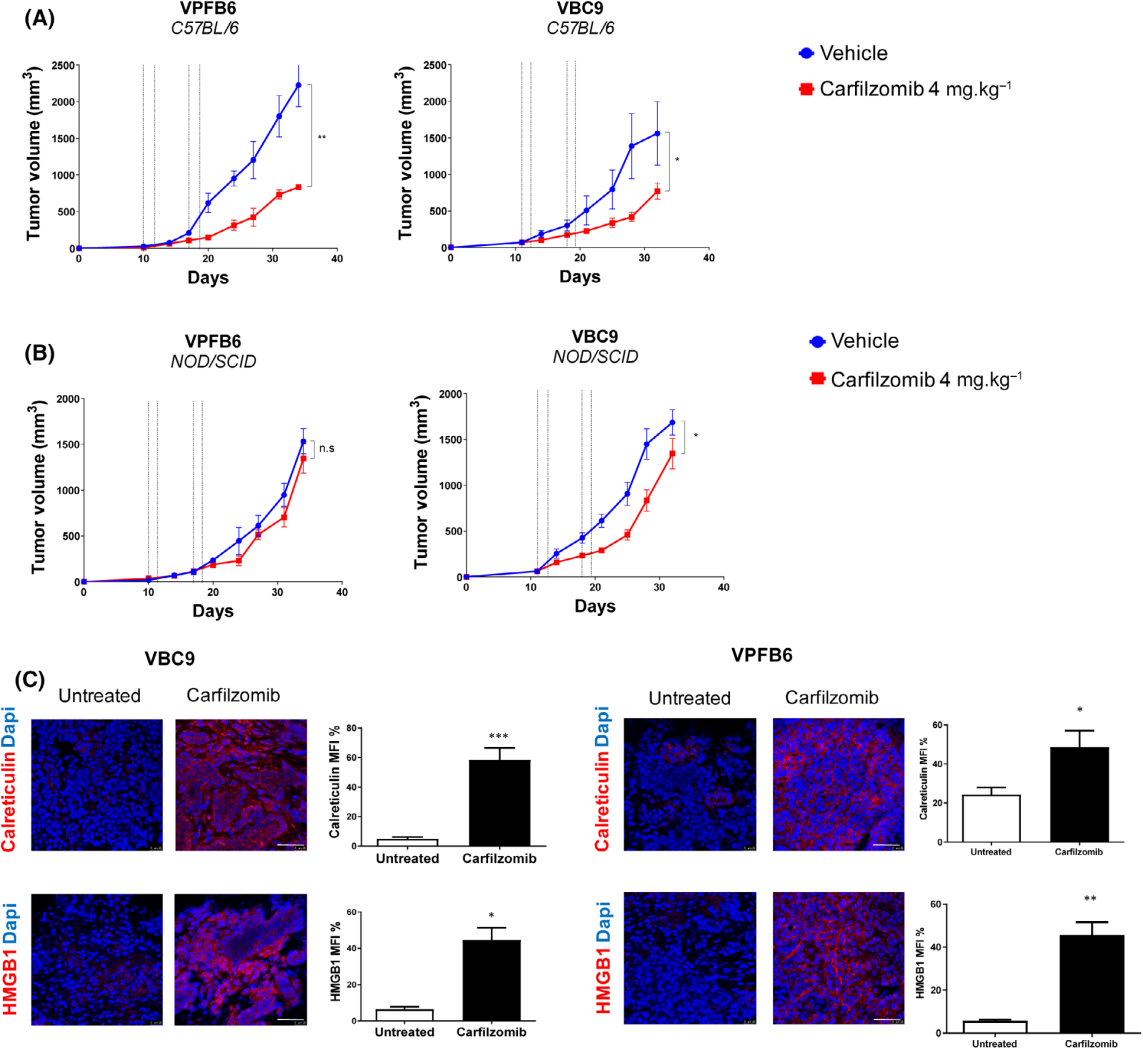
Carfilzomib impairs growth and modulates ICD markers in *BRAF* mutant murine colorectal tumors. (A) Immunocompetent (C57BL/6) mice (*n* = 10/group) were subcutaneously injected with either VPFB6 or VBC9 cells (2 × 10^6^ cells per mouse). After tumor appearance, mice were treated intravenously with vehicle (PBS) or carfilzomib (4 mg·kg^−1^) twice a week. (B) Immunodeficient (NOD/SCID) mice were subcutaneously injected with either VPFB6 or VBC9 cells (2 × 10^6^ cells per mouse). Animals bearing palpable tumors were treated as described above (*n* = 10/group). Dashed vertical lines in A and B graphs represent the times of carfilzomib administration, which was given intravenously two times per week, for two consecutive days. (C) Confocal analysis of calreticulin and HMGB1 expression in carfilzomib‐treated mice and controls. Images are representative of 5 fields per tumor sample. Bar graphs indicate confocal microscopy quantification. Mean ± SEM is displayed. Statistical significance was calculated by the Mann–Whitney *U* test. *P < 0.05, **P < 0.01, ***P < 0.001. Scale bars: 50 μm.

We set to establish whether and how the above *in vitro* findings could be transferred to *in vivo* models of *BRAF* mutant colorectal tumors. To this end, we subcutaneously injected VBC9 and VPFB6 cells in both immunocompetent (C57BL/6) and immunodeficient (NOD/SCID) mice. Carfilzomib treatment significantly inhibited tumor growth in immunocompetent animals (Fig. 4A), whereas its efficacy was mostly impaired when cancer cells were grown in immunodeficient mice, which lack mature T lymphocytes (Fig. 4B). To assess whether carfilzomib could modulate DAMPs in murine tumors, we performed *ex vivo* immunofluorescent analysis of calreticulin and HMGB1 expression. We found that both calreticulin and HMGB1 were overexpressed in carfilzomib‐treated samples (Fig. 4C). These results suggest that carfilzomib triggered the expression of danger signals, which could contribute to its antitumor activity in the context of a fully competent immune system.

### 3.5 Carfilzomib recruits T lymphocytes and NKs in *BRAF* mutant colorectal tumors

**Fig. 5:**
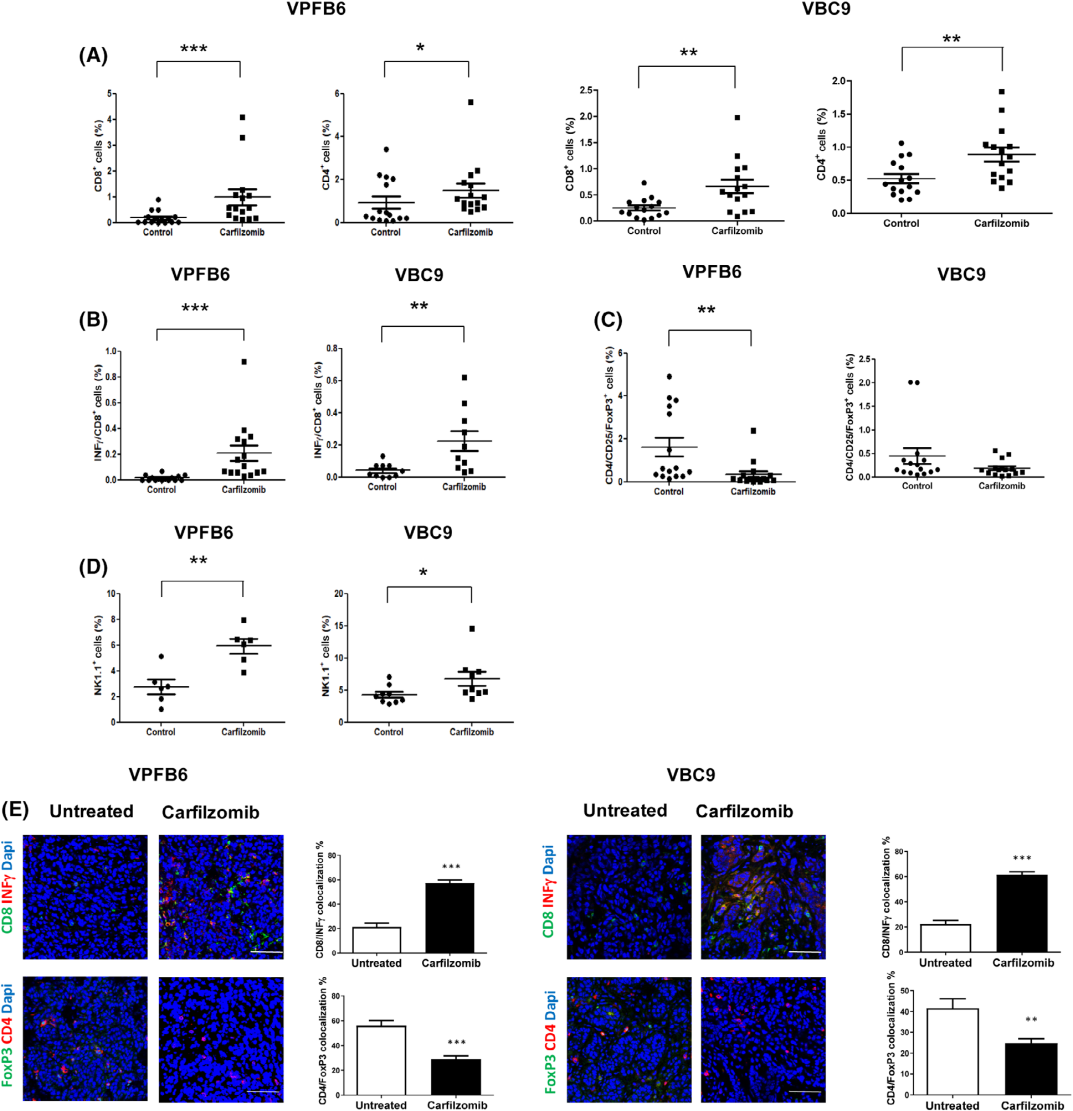
Carfilzomib enhances T lymphocyte and NK recruitment and decreases Tregs in the microenvironment of subcutaneous *BRAF* mutant murine CRCs. (A) Flow cytometry analysis of VBC9 and VPFB6 tumors freshly sacrificed at the endpoint of 2‐week treatment with carfilzomib. The number of CD4^+^ and CD8^+^ cells of each tumor is shown in the scatter plot. (B) CD8^+^/INFγ^+^, (C) CD4^+^/FoxP3^+^, (*n* = 15/group. Individual plots are presented in Fig. S4) and (D) NK 1.1^+^ (*n* = 10/group) subpopulations from carfilzomib‐treated VPFB6 and VBC9 tumors and controls are shown in the scatter plots. At least 10 mice for each treatment group were analyzed. (E) Confocal analysis showing the localization of INFγ‐producing T lymphocytes and Tregs in VPFB6 and VBC9 tumors. Bar graphs show the quantification of colocalized CD8/INFγ and CD4/FoxP3. Images are representative of 5 fields per mouse. Data and error bars indicate mean ± SEM. *P* values were calculated using by the Mann–Whitney *U* test. *P < 0.05, **P < 0.01, ***P < 0.001 Scale bars: 50 μm.

To test whether carfilzomib could reshape the tumor immune microenvironment, we performed cytofluorimetric analysis on VBC9 and VPFB6 tumors grown subcutaneously in immunocompetent animals. We found that carfilzomib induced a significant increase of both CD8^+^ and CD4^+^ cells compared with controls (Fig. 5A; see representative flow cytometry plots in Fig. S4). Next, to test whether the increased number of CD8^+^ T cells seen in carfilzomib‐treated mice corresponded to higher levels of activated cytotoxic lymphocytes, we employed interferon γ (INFγ) as a marker of T cell activation. We found increased INFγ staining in CD8^+^ cells from carfilzomib‐treated tumors (Fig. 5B). On the other hand, we also employed CD4, CD25 and FoxP3 antibodies to identify the T regulatory cell subpopulation (Tregs), which is the major determinant of immune tolerance. We observed a decrease of Tregs in carfilzomib‐treated tumors in comparison with controls (Fig. 5C). We also found a significant increase of NK cells both in VBC9 and in VPFB6 tumors treated with carfilzomib (Fig. 5D). Finally, to visualize the intratumoral localization of T lymphocytes previously identified by flow cytometry, we performed immunofluorescence analysis. We found that treatment with carfilzomib promoted activated the intratumor infiltration of T cells, as assessed by the co‐staining between CD8 and INFγ in VBC9 and VPFB6 models. We also found that the ratio between CD8^+^ T lymphocytes over CD4^+^FoxP3^+^ regulatory was higher in the carfilzomib‐treated tumors (Fig. 5E). Taken together, these results reveal that carfilzomib triggers the intratumor recruitment of activated cytotoxic T cells and Natural killers concomitant with the downregulation of Tregs, thus impairing the growth of *BRAF* mutant murine CRCs.

### 3.6 Carfilzomib impairs tumor growth and triggers CD8 T cell activation in an orthotopic model of CRC

**Fig. 6:**
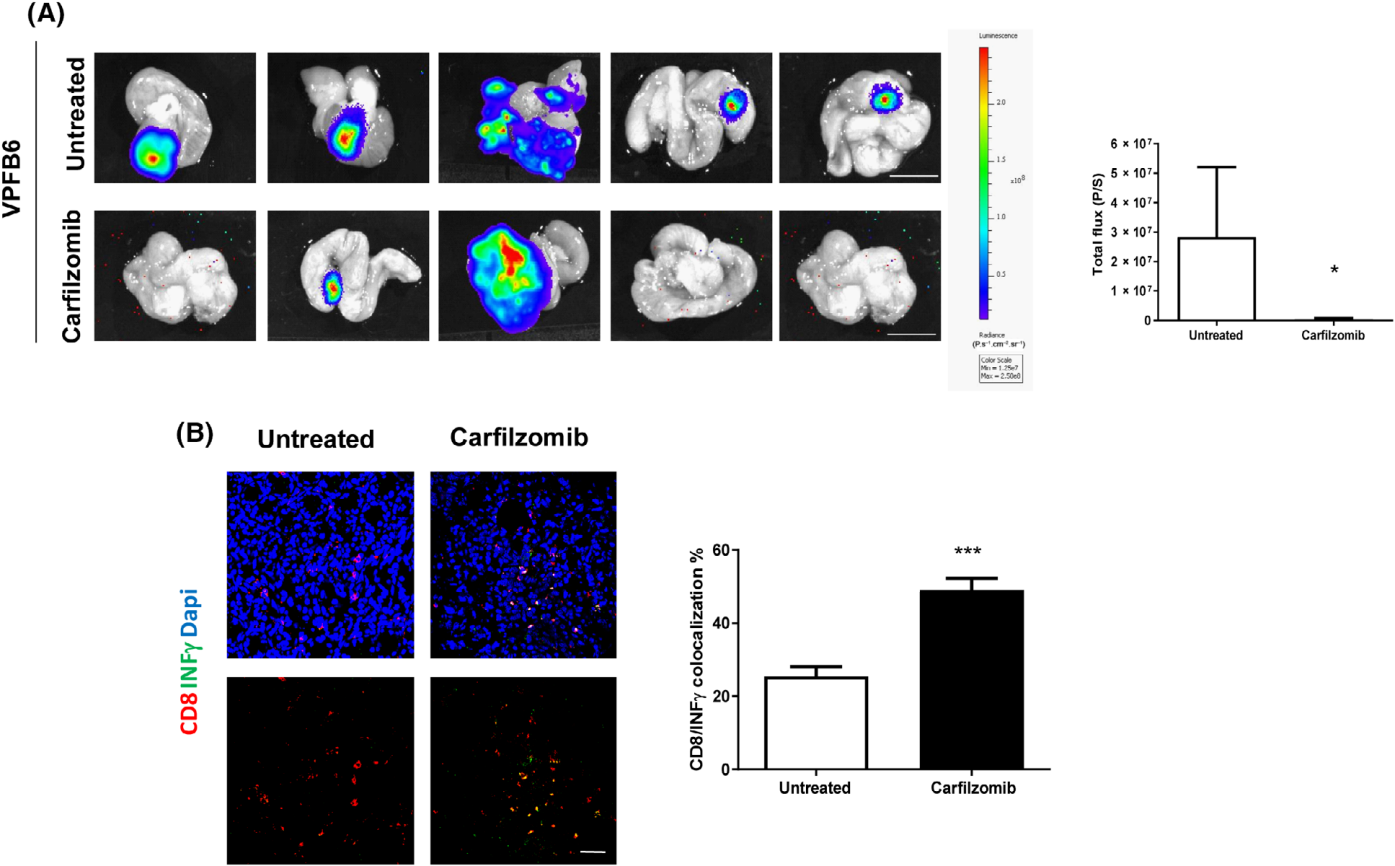
Carfilzomib inhibits tumor growth and modulates the tumor immune microenvironment in an orthotopic mouse model of CRC. (A) VPFB6 cells infected with a CMV‐Luc vector were orthotopically inoculated in the cecum of C57Bl/6 mice (*n* = 5). After 6 weeks, treatment with carfilzomib was started (4 mg·kg^−1^ twice a week, for 4 weeks). At the endpoint, mice were subcutaneously inoculated with 15 mg·mL^−1^ luciferin 5 min before the sacrifice and their guts were surgically excised. Luciferin bioluminescence was recorded through IVIS Lumina II apparatus. Bar graph shows the bioluminescence of tumor cells as total flux. Scale bars: 0.5 cm. (B) Confocal images are representative of CD8^+^/INFγ^+^ co‐staining and quantification displayed in bar graph indicates an increase of activated CD8^+^ cells after carfilzomib as compared with controls (*n* = 5 fields per mouse). Data and error bars indicate mean ± SEM. Mann–Whitney U test, two tailed: *P < 0. ***P < 0.001. Scale bars: 50 μm.

To further study the impact of carfilzomib treatment on the microenvironment of CRC, we orthotopically injected VPFB6 cells in the cecum of C57Bl/6 mice. For this purpose, we employed luciferase‐infected VPFB6 cells to monitor tumor growth by *in vivo* bioluminescence. After tumors were established, carfilzomib was administered for 4 weeks. As shown in Fig. 6A, a significant decrease of tumor growth was seen in carfilzomib‐treated mice compared with the untreated group. By immunofluorescence analysis of VPFB6 orthotopic tumors, we observed that carfilzomib promoted the intratumor infiltration by activated T lymphocytes, as assessed by CD8 and INFγ co‐localization (Fig. 6B). Unfortunately, luciferase‐VBC9 cells were rejected when injected orthotopically in immunocompetent mice, and therefore, we were not able to carry out the same experiment in this model. These results further support the evidence that carfilzomib delays tumor growth through the activation of CD8^+^ T cells, which are the main effectors of antitumor immune surveillance.

## 4 Discussion

The proteasome inhibitor carfilzomib impairs the recycling of misfolded proteins, leading to ER stress and cell death. Over the past 20 years, inhibition of the proteasome chymotrypsin‐like activity has shown clinical efficacy in patients with hematological malignancies [39]. More recently, a preclinical work has provided evidence that carfilzomib may have activity also in solid tumors [40].

Treatment of mCRC patients bearing *BRAF* mutant tumors remains challenging. In particular, *BRAF* mutant colorectal tumors have a more aggressive phenotype, an immune suppressive microenvironment and lower clinical response to conventional cytotoxic agents and immunotherapy. Our previous screening efforts in isogenic colon cancer cells revealed a collateral vulnerability to proteasome inhibitors of cells carrying the *BRAF* V600E mutation [24]. In the current work, we established the antitumor activity of carfilzomib in several human and murine *BRAF* mutant CRC models. Our preclinical study dissected the possible mechanisms by which carfilzomib exerts a cell‐autonomous and a non‐cell‐autonomous antitumor effect by modulating the tumor immune microenvironment. In this regard, we confirmed that carfilzomib directly kills human and murine *BRAF* mutant CRC cells *in vitro* in a dose‐dependent manner. We also found that carfilzomib induced ER stress and autophagy in human and murine *BRAF* mutant CRC cells. This finding is in line with previous works reporting that carfilzomib increased CHOP protein levels and induced ER stress in chronic lymphocytic leukemia and multiple myeloma [41, 42].

ER stress and autophagy have been shown to be part of the so‐called ‘integrated stress response’ leading to the release of several effector molecules, defined as DAMPs that may be sensed by specific receptors on immune cells [43, 44]. Previous studies on multiple myeloma cells indicated that carfilzomib induce the activation of DAMP pathways [45]. We found that carfilzomib promotes the release of ATP, HMGB1 and ANXA1 in the cell supernatant and calreticulin translocation on the surface of *BRAF* mutant CRC cells *in vitro*. These findings are in agreement with previous works showing that carfilzomib could sustain the unfolded protein response and the *in vitro* mobilization of HMGB1 and calreticulin in multiple myeloma cells [20].

The emission of DAMPs, occurring in response to several chemotherapeutic drugs, is the result of the interconnection of molecular events, leading to antigen‐specific immune responses and immunological memory [46, 47]. However, not all cytotoxic agents are able to unleash DAMPs, and ICD inducers cannot be identified based on structural or functional similarities. While artificial intelligence algorithms have been used to predict the ability of specific molecules to promote ICD based on their physicochemical properties, *in vivo* experiments are required to formally validate such predictions [48]. For instance, among platinum‐based chemotherapeutic drugs, only oxaliplatin is able to trigger ICD in preclinical settings [8], while among proteasome inhibitors, bortezomib is known to induce ICD across several tumor types [29, 49, 50]. On the contrary, the effects of carfilzomib on the microenvironment of solid tumors are less characterized. It has been reported that carfilzomib potentiates immune checkpoint therapy in a lung cancer model by reprogramming tumor‐associated macrophages into M1‐like macrophages [51]. Here, we have shown that carfilzomib mobilizes ICD markers and exerts anticancer activity in *BRAF* mutant CRC mouse models. Indeed, carfilzomib delayed tumor progression in the presence of a competent immune system, while immunodeficient mice derived only little or no benefit from treatment. These results obtained in both subcutaneous and orthotopic mouse models provide evidence that carfilzomib elicits an immune‐mediated response against *BRAF* mutant CRCs.

The emission of DAMPs in carfilzomib‐treated tumors and the requirement of an intact immune system for carfilzomib to exert its antitumor activity *in vivo* are clues that this proteasome inhibitor may act as an ICD inducer in *BRAF* mutant colorectal tumors. However, additional experiments including a vaccination assay with carfilzomib are needed to clarify its exact mode of action.

We acknowledge that the preclinical efficacy of carfilzomib in immunocompetent mice was still suboptimal as we did not observe any complete tumor regression. This finding could be related to multiple factors concerning the pharmacokinetic and the pharmacodynamic of this proteasome inhibitor. *In vitro* experiments clearly indicate that carfilzomib exerts its cytotoxic effect on *BRAF* mutant CRC cells in a dose‐dependent manner, and we obtained a complete cell growth inhibition only at the higher concentrations, which may not be reachable intratumorally when the drug is administered intravenously to mice. A second possible explanation of the limited *in vivo* cytotoxicity of carfilzomib can be related to the cytoprotective effect of autophagy, which can promote a survival response enabling tumor cells to overcome apoptotic signals [38, 52]. We speculate that autophagy may allow tumor cells to tolerate treatment and recover during the interval between chemotherapy cycles *in vivo*. Finally, it is also possible that proteasome inhibition by carfilzomib could impair antigen presentation by cancer cells, thus balancing off some of its beneficial effects on the reshaping of the tumor immune microenvironment. Aberrations in the so‐called “antigen processing and presenting machinery” have been frequently observed in human tumors [15]. The majority of class I (MHCI) peptides are proteasome degradation products of cytosolic proteins that are transported into the endoplasmic reticulum by transporters associated with antigen processing. Proteasome inhibition can interfere with peptide presentation on MHC class I molecules. It has also been observed that the autophagy machinery elicited by proteasome inhibitors may have a negative impact on MHCI antigen cross‐presentation by dendritic cells [53]. Therefore, we speculate that, from one side dying cells upon carfilzomib treatment emit danger signals with immunostimulant effect, while on the other side, proteasome inhibition could repress the antigen presenting machinery making cancer cells less attractive for killing by the immune cells. However, its immunomodulant properties render carfilzomib an attractive drug for novel and more efficient combination therapies.

The majority of patients with microsatellite stable *BRAFV600E* CRC are refractory to immunotherapeutic regimens. Several therapeutic strategies have been developed for the treatment of these metastatic colorectal patients. For instance, an ongoing clinical study (NCT04017650) on unresectable *BRAFV600E* mutant tumors is exploring the potential efficacy of the treatment with encorafenib and cetuximab in combination with immunotherapy with monoclonal antibodies, such as nivolumab. In this regard, despite the above limitations, our study extensively characterizes for the first time the ability of an FDA‐approved drug to modulate the immunosuppressive microenvironment of BRAF mutant MSS colorectal tumors in mice. Carfilzomib induced significant changes in the composition of the tumor immune infiltrate by recruiting activated T and NK cells. The increased ratio of cytotoxic CD8^+^ T lymphocytes over FOXP3^+^ regulatory T cells within the tumor upon chemotherapy predict favorable therapeutic responses in many cancer types [54, 55]. For instance, CD8^+^ tumor‐infiltrating lymphocytes are associated with favorable prognosis in patients with triple‐negative breast cancer [56] and non‐small cell lung cancer [57]. Interestingly, in breast or CRC patients treated with the ICD inducers anthracyclines and oxaliplatin, respectively, increased cytotoxic CD8^+^ T lymphocytes resulted crucial for the outcomes of therapy [58]. In our work, we observed that carfilzomib induces a reshape of the tumor microenvironment mediating the recruitment of activated of CD8^+^ T lymphocytes and decreasing the amount of CD4^+^/FOXP3^+^ regulatory T cells. Of note, it has been demonstrated that the evaluation of cytotoxic and regulatory T cell spatial distribution is equally important. [59]. By confocal analysis on tissue specimens, we clearly visualized the stromal distribution of CD8^+^ lymphocytes along with NK cells, which are crucial to initiate an adaptive response [60].

Intratumoral recruitment of cytotoxic immune cells is relevant for effective cancer immunotherapy. In the last decade, immunotherapy and in particular, immune checkpoint inhibitors have been approved for the treatment of several tumors. However, intrinsic unresponsiveness occurs in many cases. In this regard, intense research efforts are being deployed to identify strategies capable of improving cancer cell susceptibility to immune modulator agents. Of relevance to our study, we report that a phase I clinical trial is testing carfilzomib and nivolumab together with pelareorep and dexamethasone for the treatment of patients with multiple myeloma (ClinicalTrials.gov Identifier: NCT03605719). However, no clinical studies have been designed to explore combinatorial regimens of carfilzomib and immunotherapy in solid tumors.

## 5 Conclusions

In summary, we provided direct evidence that carfilzomib exerts a cytotoxic effect on both human and murine *BRAF* mutant cells. In addition, carfilzomib induces an accumulation of misfolded proteins, leading to ER stress and, in turn promoting to the mobilization of immunostimulant danger signals, including calreticulin exposure and HMGB1, ATP and ANXA1 release. Therefore, we demonstrated herein that carfilzomib antitumor effect is enhanced by an indirect tumor immune microenvironment modulation. Together our results provide new insights on the ability of carfilzomib in stimulating anticancer adaptive immunity in *BRAF* mutant CRC mouse models, thus supporting the clinical development of combination treatments with carfilzomib and immunomodulating agents.

## Conflict of interest

FDN has received speaker's fees from Illumina and Pierre Fabre outside of the submitted work. AB reports receipt of grants/research supports from Neophore, AstraZeneca and Boehringer, and honoraria/consultation fees from Guardant Health, Inivata. AB is stock shareholder of Neophore and Kither. AB is advisory boards member for Inivata, Neophore, Roche/Genentech. M Massaia reports advisory boards for AbbVie, Janssen‐Cilag, Sanofi, and research funding from Sanofi outside of the submitted work.

## Author contributions

FDN, FM and DO conceived the project. FDN, FM, AB, DO, RR, DS, M Massaia and LB designed the experiments. RR, DS and CR provided key reagents. FM, DO, FG, CF, MP, M Macagno, VP, CG, AM, and BC performed the experiments. FM, DO, FDN, AB, GC, and SL analyzed the data. FDN, AB, FM, DO, LB, DS, CF and RR interpreted the results. FDN, FM, DO wrote the manuscript. All authors read and approved the manuscript.

### Peer review

The peer review history for this article is available at https://www.webofscience.com/api/gateway/wos/peer‐review/10.1002/1878‐0261.13595.

## Supporting information


**Fig. S1.** Carfilzomib inhibits proteasome activity and induces poly‐ubiquitinated protein accumulation. (A) Colorectal cancer (CRC) cells were exposed to carfilzomib (100 nM) for 1, 2, 4, 6, 16 or 24 h and then assayed using Proteasome‐Glo Chymotrypsin‐Like Cell‐Based Assay (Promega) to test proteasome activity, compared to the untreated cells. The graphs show mean values of proteasome activity ±SEM of two independent experiments. Statistical differences were determined with the Mann–Whitney *U* test. *P < 0.05, **P < 0.01. (B) Western blot analysis showing the amount of poly‐ubiquitinated proteins in *BRAF* mutant CRC cell lines. Cells were cultured for 24 h in the presence or absence of carfilzomib (100 nM). Vinculin detection was used as loading control.

**Fig. S2.** Representative analysis by flow cytometry of Annexin V/Propidium Iodide positive events in human (WiDr, HROC24, KP363T and JVE127) and murine (VBC9 and VPFB6) *BRAF* mutant colorectal cancer cells.

**Fig. S3.** Autophagy induced by carfilzomib acts as a cytoprotective mechanism in *BRAF* mutant colorectal cancer (CRC) cells. The proliferation of carfilzomib‐treated cells was evaluated by colony‐forming assay; human (WiDr, KP363T and JVE127) and murine (VBC9 and VPFB6) *BRAF* mutant CRC cells were treated with carfilzomib, chloroquine or their combination. Chloroquine was used at the fixed concentration of 3 μM, while carfilzomib concentration was selected for each cell line based on their drug sensitivity and employed as following: WiDr – 5 nM; KP363T – 5 nM; JVE127–10 nM; VBC9–5 nM; VPFB6–10 nM. Dimethyl sulfoxide (DMSO) treated cells were used as proliferation control. The experiment was replicated at least twice.

**Fig. S4.** Flow cytometry analysis of VBC9 and VPFB6 tumors freshly collected after two weeks of carfilzomib treatment. The number of T helper cells (CD4^+^) and cytotoxic T cells (CD8^+^) and natural killer (NK 1.1) of each tumor from single carfilzomib‐treated mouse or control is shown in the relative scatter plots. Positive cells were calculated on CD45^+^ (protein tyrosine phosphatase receptor type C) CD3^+^ (T cell lineage marker) live events. Similarly, the percent of activated cytotoxic CD8^+^T cells/INFγ^+^ (interferon γ), and T regulatory cells, assessed by CD4^+^ and FoxP3^+^ (forkhead box protein P3) markers, has been evaluated.

**Table S1.** Short tandem repeat (STR) profiles of human cancer cell lines are listed.

**Table S2.** Short tandem repeat (STR) profiles of mouse cancer cell lines are listed.

## Data Availability

The data that supports the findings of this study are available in the accompanying figures and supplementary data. The STR profiling of human *BRAF* mutant colorectal cancer cells is listed in Table S1. The STR profiling of murine *BRAF* mutant colorectal cancer cells are listed in Table S2. The molecular characterization of murine *BRAF* mutant colorectal cancer cells derives from public domain resources: Rad, R., et al. A genetic progression model of Braf (V600E)‐induced intestinal tumorigenesis reveals targets for therapeutic intervention. Cancer cell 24, 15–29 (2013), at DOI:10.1016/j.ccr.2013.05.014.
