# Supplementary figures and images for: Preclinical efficacy of carfilzomib in BRAF‐mutant colorectal cancer models
Source: Mol Oncol. 2024 Feb 13;18(6):1552–70. doi: 10.1002/1878-0261.13595 (PMC11161726; doi:10.1002/1878-0261.13595)

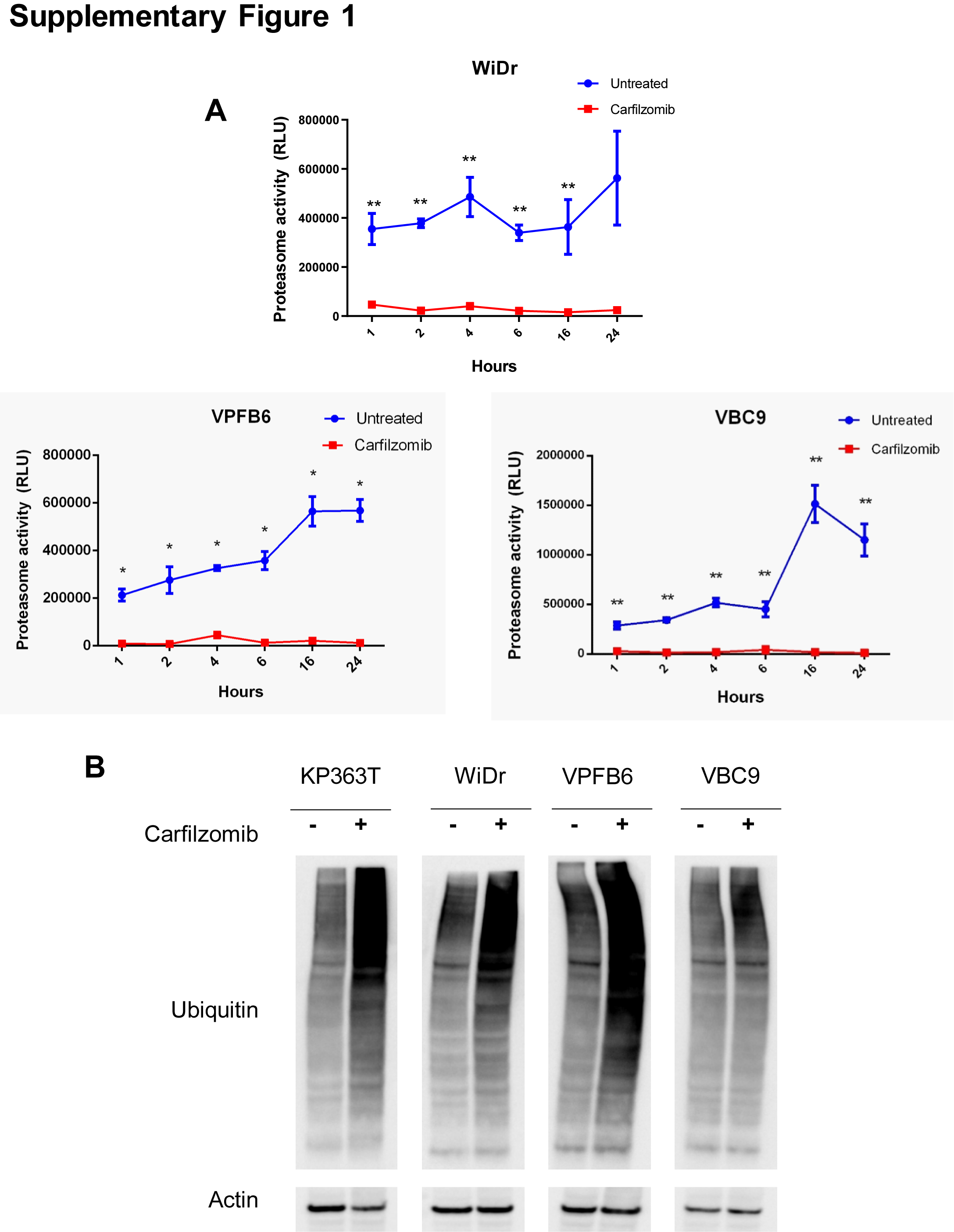

Supplement: Supplementary file 1 — Fig. S1. Carfilzomib inhibits proteasome activity and induces poly‐ubiquitinated protein accumulation. (A) Colorectal cancer (CRC) cells were exposed to carfilzomib (100 nM) for 1, 2, 4, 6, 16 or 24 h and then assayed using Proteasome‐Glo Chymotrypsin‐Like Cell‐Based Assay (Promega) to test proteasome activity, compared to the untreated cells. The graphs show mean values of proteasome activity ±SEM of two independent experiments. Statistical differences were determined with the Mann–Whitney U test. *P < 0.05, **P < 0.01. (B) Western blot analysis showing the amount of poly‐ubiquitinated proteins in BRAF mutant CRC cell lines. Cells were cultured for 24 h in the presence or absence of carfilzomib (100 nM). Vinculin detection was used as loading control. [file MOL2-18-1552-s005.tif]

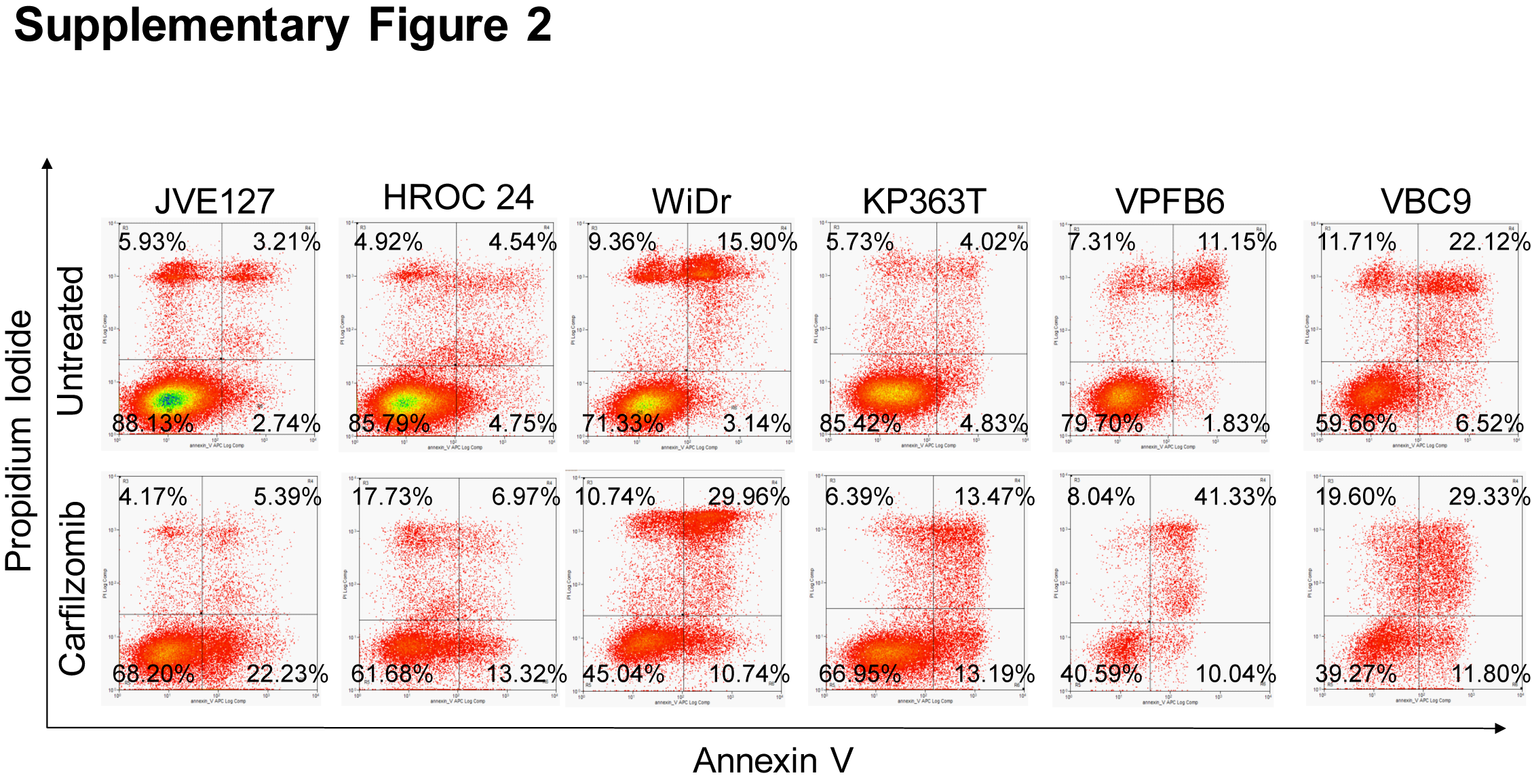

Supplement: Supplementary file 2 — Fig. S2. Representative analysis by flow cytometry of Annexin V/Propidium Iodide positive events in human (WiDr, HROC24, KP363T and JVE127) and murine (VBC9 and VPFB6) BRAF mutant colorectal cancer cells. [file MOL2-18-1552-s001.tif]

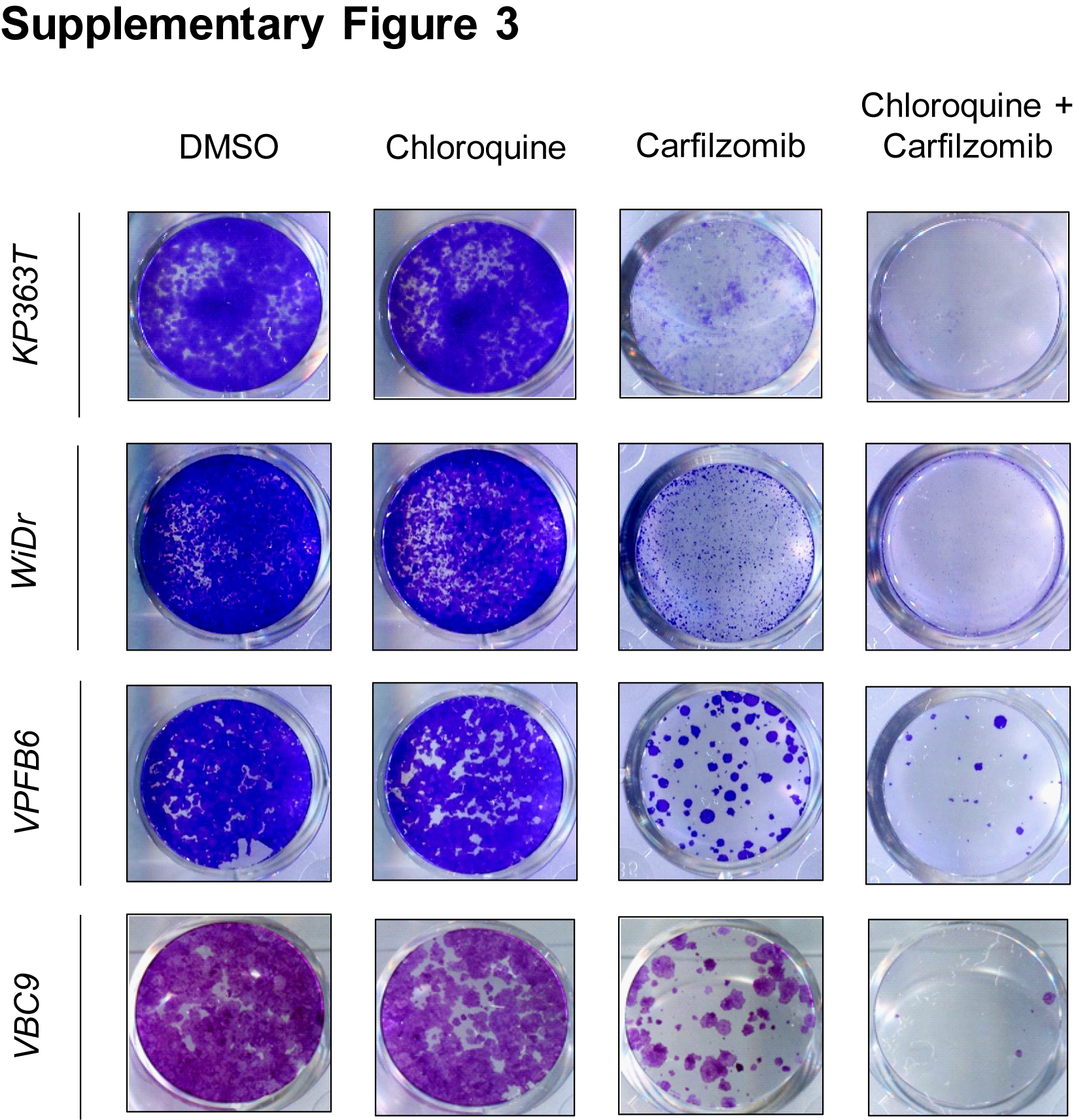

Supplement: Supplementary file 3 — Fig. S3. Autophagy induced by carfilzomib acts as a cytoprotective mechanism in BRAF mutant colorectal cancer (CRC) cells. The proliferation of carfilzomib‐treated cells was evaluated by colony‐forming assay; human (WiDr, KP363T and JVE127) and murine (VBC9 and VPFB6) BRAF mutant CRC cells were treated with carfilzomib, chloroquine or their combination. Chloroquine was used at the fixed concentration of 3 μM, while carfilzomib concentration was selected for each cell line based on their drug sensitivity and employed as following: WiDr – 5 nM; KP363T – 5 nM; JVE127–10 nM; VBC9–5 nM; VPFB6–10 nM. Dimethyl sulfoxide (DMSO) treated cells were used as proliferation control. The experiment was replicated at least twice. [file MOL2-18-1552-s002.tif]

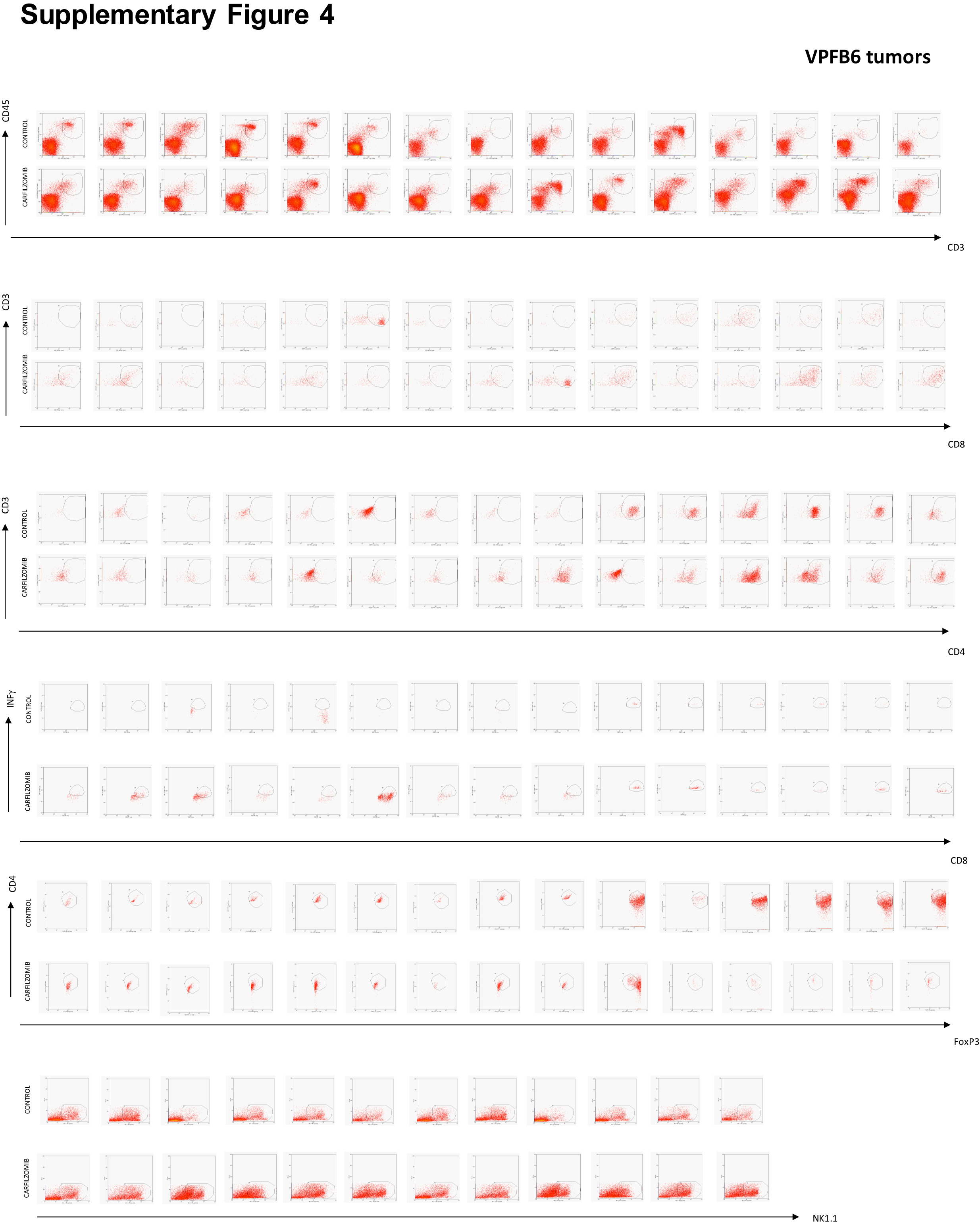

Supplement: Supplementary file 4 — Fig. S4. Flow cytometry analysis of VBC9 and VPFB6 tumors freshly collected after two weeks of carfilzomib treatment. The number of T helper cells (CD4+) and cytotoxic T cells (CD8+) and natural killer (NK 1.1) of each tumor from single carfilzomib‐treated mouse or control is shown in the relative scatter plots. Positive cells were calculated on CD45+ (protein tyrosine phosphatase receptor type C) CD3+ (T cell lineage marker) live events. Similarly, the percent of activated cytotoxic CD8+T cells/INFγ+ (interferon γ), and T regulatory cells, assessed by CD4+ and FoxP3+ (forkhead box protein P3) markers, has been evaluated. [file MOL2-18-1552-s003.zip › Maione_Oddo_Suppl_Fig 4 b.tif]

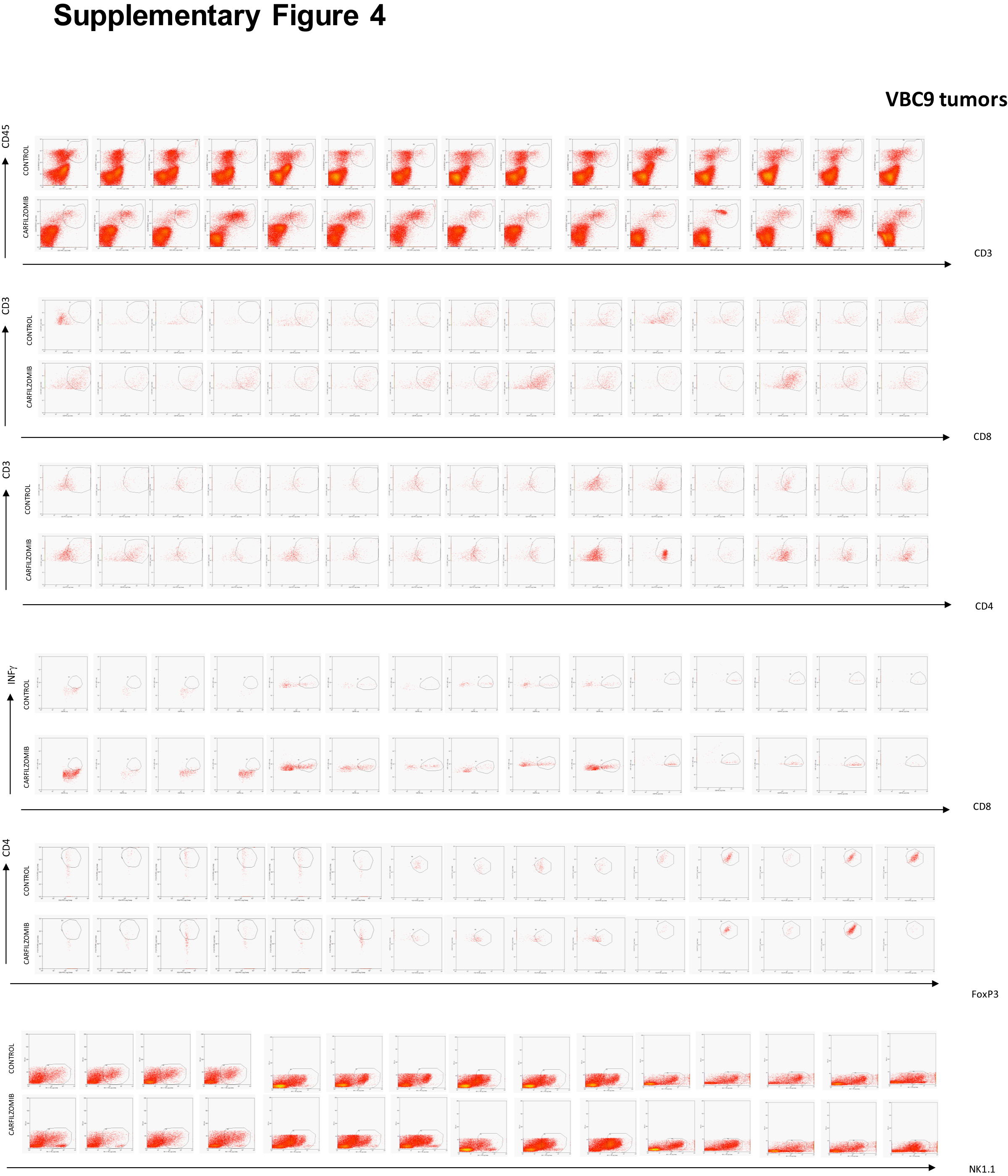

Supplement: Supplementary file 4 — Fig. S4. Flow cytometry analysis of VBC9 and VPFB6 tumors freshly collected after two weeks of carfilzomib treatment. The number of T helper cells (CD4+) and cytotoxic T cells (CD8+) and natural killer (NK 1.1) of each tumor from single carfilzomib‐treated mouse or control is shown in the relative scatter plots. Positive cells were calculated on CD45+ (protein tyrosine phosphatase receptor type C) CD3+ (T cell lineage marker) live events. Similarly, the percent of activated cytotoxic CD8+T cells/INFγ+ (interferon γ), and T regulatory cells, assessed by CD4+ and FoxP3+ (forkhead box protein P3) markers, has been evaluated. [file MOL2-18-1552-s003.zip › mol213595-sup-0004-FigureS4.tif]

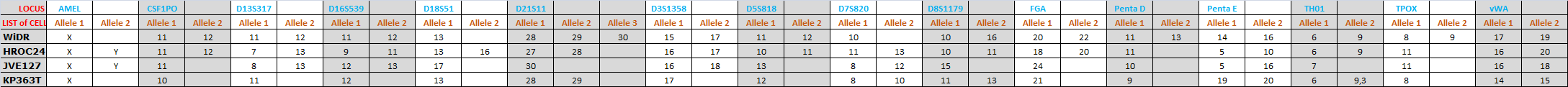

Supplement: Supplementary file 5 — Table S1. Short tandem repeat (STR) profiles of human cancer cell lines are listed. [file MOL2-18-1552-s006.tif]

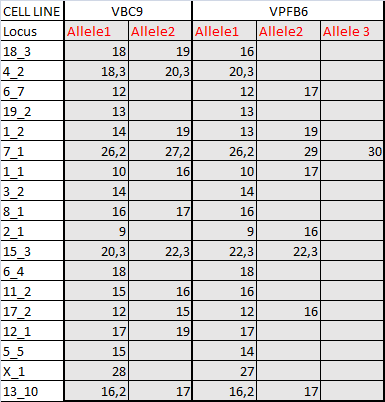

Supplement: Supplementary file 6 — Table S2. Short tandem repeat (STR) profiles of mouse cancer cell lines are listed. [file MOL2-18-1552-s004.tif]
